# Co-creating inclusive spaces and places: Towards an intergenerational and age-friendly living ecosystem

**DOI:** 10.3389/fpubh.2022.996520

**Published:** 2023-01-04

**Authors:** Mei Lan Fang, Judith Sixsmith, Alison Hamilton-Pryde, Rayna Rogowsky, Pat Scrutton, Ro Pengelly, Ryan Woolrych, Rachel Creaney

**Affiliations:** ^1^School of Health Sciences, University of Dundee, Dundee, United Kingdom; ^2^Department of Gerontology, Science and Ageing Technology (STAR) Research Institute, Simon Fraser University, Vancouver, BC, Canada; ^3^The Urban Institute, Heriot Watt University, Edinburgh, United Kingdom; ^4^Intergenerational National Network, Scotland, United Kingdom; ^5^ScotSectorlink, Scotland, United Kingdom; ^6^The James Hutton Institute, Dundee, United Kingdom

**Keywords:** lifespan, age-friendly cities and communities, ecological theory, co-creation, creative methods, sustainable development, transdisciplinary working

## Abstract

**Introduction:**

Evolving aging societies, ongoing digitalisation and circumstances of COVID-19 are changing living conditions for growing older. There is an increased urgency to view public health with a focus on integrating people of all ages into the matrix of opportunities afforded in their communities. This study initiates the conceptualization of an intergenerational, age-friendly living ecosystem (AFLE) to enhance public health planning.

**Methodology:**

A participatory study was conducted using a multi-methods approach. Six virtual co-creation sessions (*n* = 35–50 participants), alongside a mainly open-ended INTERGEN survey designed specifically for this study (*n* = 130) were conducted to conceptualize multilevel ideas for building intergenerational age-friendly places using Bronfenbrenner's ecological systems model. At the height of COVID-19, virtual applications (Zoom, Moodboard) and case studies, creative methods (drawing, photography, storytelling and spotlight sessions) were applied to engage academic and non-academic participants between ages 5 – 80+ years, across eight countries. Sessions were video-recorded with visual themes captured by a graphic facilitator. The survey covered issues of multigenerational interactions; intergenerational and age-friendly place features; place safety; and necessary stakeholders required for creating intergenerational and age-friendly places. Data were reflexively analyzed using a team approach to thematic analysis.

**Results:**

Findings present both the thematic analysis of Virtual Co-creation Camps (VCCs) and the INTERGEN survey results. These findings are addressed in three overarching categories that highlight the necessary characteristics of AFLEs as suggested by the VCC participants and survey respondents: (i) Sensory factors: feeling and emotion as starting points for physical design; (ii) Physical and digital factors in designing AFLE spaces and places; and (iii) Socio-cultural factors: tackling ageism and exclusion as part of the solution.

**Discussion:**

The analysis resulted in a pathway toward enhanced understandings on how multi-generations can better interact with fluctuating organizational domains (industry, voluntary, academic and public sectors) in urban and rural settings to facilitate intergenerational connectivity. Through processes of co-creation, an AFLE proof of concept and roadmap for public health planning was developed to support and provide opportunities for people as they age to reap the socioeconomic benefits of their local and virtual communities and help them become well integrated, valued and contributory members of society.

## 1. Introduction

At present, there are ~962 million people over the age of 60 worldwide (1). By 2050, this figure is predicted to double. Population aging combined with rapid urbanization and increasing digitization of social spaces and resources are social changes which have created global challenges. At the micro level, some older people are becoming more age-segregated and at risk of social, economic and digital exclusion (2, 3). At the meso level, the role of older individuals within family, social, community and employment spheres is often undervalued and poorly supported (2, 3). On a broader macro scale, concomitant age-related physical and mental health conditions (shaped by loneliness and social isolation) has meant that care provision has become more expensive and less manageable across societies (2, 3). Such social and care transformations highlight the need for cities and communities to modify existing structures and services toward a more integrated and inclusive system that provides social and economic opportunities (e.g., employment, education and skills development, access to information and formal / informal social participation) for people of all ages (4, 5). Indeed, the need for better ways to involve older people within communities and in societies (as a whole) became acutely apparent throughout the COVID pandemic—demonstrated by events and discourses that bring to the fore their pervasive marginalization in mainstream society (6, 7).

In recognition of the challenges and unmet needs, members of the Intergenerational National Network (INN)—a grassroots organization based in Scotland—began exploring possibilities for co-creating more sustainable and inclusive intergenerational communities *with and for* people of all ages and cultures to: reduce social distance between groups and across generations; enhance social and economic opportunities through intergenerational knowledge exchange; and enable individuals who are reported to frequently experience loneliness and isolation (such as people living with dementia) to remain integrated and connected with their communities. Although the initiative was established in Scotland, members of the INN recognize that the aforementioned challenges are occurring worldwide, and it was important to include ideas and promising practices from across geographic and cultural contexts.

Through seed funding provided by the Scottish University Insight Institute (SUII), international collaborations and co-creation activities were undertaken as part of a trans-national co-creation study. At the height of COVID-19 in May 2020, older people, students and academics across disciplines (health sciences, urban studies, materials design, gerontology, social work, psychology, education); and professionals in various sectors (architecture, urban planning, health, education and voluntary/third sector) from eight countries (Scotland, Denmark, China, India, Slovakia, Singapore, Canada, Australia), were brought together on a virtual platform to co-create research, policy and practice ideas and concepts toward developing an intergenerational, age-friendly community ecosystem (AFLE). It is important to note that given that this is a co-creation study focused on knowledge co-creation and exchange, the purpose of bringing together transnational stakeholders is not to enable cross-cultural comparability but rather to harness important and varied cross-cultural knowledges to co-produce solutions with the potential for mutual benefit and impact.

### 1.1. The public health challenge

As Kaplan et al. (8) have argued, intergenerational integration and connectedness forms the bedrock of strong, supportive communities. According to the World Health Organization's (WHO) Global Age-friendly Cities Guide (9), “intergenerational activities are considered to be more desirable than activities for older people alone” (p. 42). However, mainstream leisure and service models are not necessarily age-friendly nor are they always well connected with specialized service provisions across generations (10). However, there is a plethora of studies that seek to incorporate intergenerational elements within existing service provision for older adults (11, 12).

While there are exemplary health service initiatives that facilitate intergenerationality (11, 12), they are generally more healthcare oriented, with perhaps less emphasis on bringing intergenerational social connected-ness through adapting existing and/or creating new social spaces and places and servicing within these (such as community cafés, community gardens, youth and seniors centers). Key challenge in public health have been to move beyond a focus on the biomedical model and resources invested to determine and understand etiology of disease and illness, toward preventing, tracking and monitoring outbreaks and primary healthcare (13, 14). Place and environmental determinants of health have been somewhat overshadowed, and within this domain, the notion of intergenerationality has only more recently been highlighted as a topic of priority through place-focused projects such as PlaceAge (15).

However, this is not to say that there is a shortage of intergenerational initiatives. In fact, intergenerational programming and efforts are prolific in local communities in the shape of less well-known grassroots intergenerational programmes (such as the Old's Cool Intergenerational Project at the Citadel Youth Center in Scotland) and initiatives (such as the AFLE project spearheaded by the INN). Hence, knowledge exchange involving brainstorming and co-creating ideas and actions between and with diverse stakeholders (i.e., from different cultures, with various life experiences, working in different sectors, trained in different disciplines), particularly those working out of grassroots organizations and across the age range is key. This project prioritized the bringing together of a range of perspectives for defining and unpacking existing theoretical and knowledge gaps for conceptualizing age-friendliness within and across spaces and places, with a focus on the services and care situated within them and how these can be enhanced for older people through intergenerationality.

### 1.2. The AFLE project: Addressing the challenge

The proposed project, in collaboration with the INN, co-created research, policy, and practice recommendations developing intergenerational, age-friendly community ecosystems. The work was informed by Kaplan et al. ideas which situate intergenerational relationships as central to healthy and productive aging—and an important component of sustainable and liveable societies (8). By uniting different generations in purposeful, equitable, and participatory activities, we can generate space for positive intergenerational connectedness where perceptions and organizational attitudes are challenged, positions and identities are questioned and reformed, and mindsets and working practices are changed.

The aim of this project was to bring together researchers, industry professionals, policymakers, health and housing practitioners, and multigenerational members of the community along with non-government organizations, universities and collaborators from the United Kingdom (UK), China, India, Canada, Denmark, Lithuania, Singapore, Australia, and Slovakia to engage in knowledge mobilization to inform public health planning and policy. The goal was to generate research ideas, and policy and practice recommendations and potential solutions regarding how diverse knowledge and resources can be pooled to make the best use of community and industry spaces to develop a living age-friendly international ecosystem of places that facilitate intergenerational working across communities and sectors. The project's aim aligns well with United Nations (UN) Sustainable Development Goals (SDGs) that are focussing on “mobilize efforts to end all forms of poverty, fight inequalities and tackle climate change, while ensuring that no one is left behind” (16). The anticipated research, policy, and outputs of our project will specifically aim to tackle SDG 3 to “ensure healthy lives and promote wellbeing at all ages” (16) and SDG 11 to make “cities inclusive and human settlements, inclusive, safe, resilient and sustainable” (16).

It is important to note that genuine participatory working to co-create opportunities for developing mutually beneficial spaces *for all* is a substantial undertaking. It requires working across disciplines and sectors as well as prioritizing community and lay perspectives in the development and decision-making process. This is particularly the case when undertaking complex participatory, people-centered research that requires input and participation from diverse disciplines and stakeholder groups—or in other words, transdisciplinary working (17).

Transdisciplinary working, according to Boger et al., is an attempt to access the *collective mind* (18), of a team composed of different viewpoints to solve a difficult real-world problem, known for the purpose of generating transformative change, as a *wicked problem* (19). Consequently, the project was conducted by an extensive group of academic, service sector, community-based groups, policymakers, and older and younger experiential stakeholders. Together, we identified a need to develop intergenerational models using co-production frameworks to inform the creation of inclusive and integrative age-friendly environments. Aligned with principles of transdisciplinary working, a community-based participatory research (CBPR) approach (grounded on principles of equity, inclusivity, empowerment, partnership working, and co-creation), and carefully selected CBPR knowledge co-creation methods were applied to stimulate in-depth knowledge exchange and transdisciplinary working across international collaborators and partners (18).

The AFLE project undertook a community-based participatory, people-centered multimethod approach that emphasized the importance of: (1) communal learning and collective knowledge co-creation; (2) development of collective efficacy through mutual affirmation; (3) the need to foster intergenerational leadership; and (4) working jointly across disciplines and sectors—transcending ideational boundaries (20). The principles of CBPR were promoted through the reciprocal transfer of knowledge and expertise; inclusive participation; power sharing and equity; and data ownership across all partners (21).

AFLE's work programme encouraged knowledge exchange by first adopting a democratization of knowledge and effective knowledge transfer (KT) strategy, recognizing that KT significantly impacts research and policy (22). The work programme prioritized seldom heard voices and enhanced participation from all stakeholders throughout the entirety of the research process: in setting the aims and objectives; conceptual development; rules of engagement during sessions, shaping the research design, policy, and practice recommendations; and also enacting *responsibilisation* to the project (23), by way of following through with project commitments and pledging to complete actions established at co-creation events.

To ensure an action-oriented process, this co-creation initiative focused on co-producing a set of outputs planned at the outset with project partners that included a:

- Strategy for the development of a culturally appropriate age-friendly, living ecosystem of intergenerational virtual spaces and built places.
- Conceptual ecosystem map of community hub ideas.
- Emergence of a community of practice in each country to spearhead their own development.
- Policy and practice road map to inform the development of an intergenerational and age-friendly living ecosystem.
- Proposal for an upscaled longitudinal research proposal for submission to national funding bodies.
- Virtual time capsule in the form of a website to track progress and impact: www.afle.co.uk.

## 2. Methodology

Informed by Kang's (24) approach to creating an intergenerational community of practice, and Boger et al. (18) notion of transdisciplinarity, the AFLE project undertook a participatory multi-methods approach, emphasizing the importance of: (i) communal learning and collective knowledge co-creation; (ii) development of collective efficacy through mutual affirmation; (iii) the need to foster intergenerational leadership; and (iv) working jointly across disciplines and sectors—transcending ideational boundaries. In terms of theory, the project was guided and operationalized according to the ecological theory, namely, Bronfenbrenner's socio-ecological systems model. Discussed in the following section are key conceptual and theoretical underpinnings of the AFLE project.

### 2.1. Conceptual and theoretical underpinnings of the AFLE

According to the WHO Age-Friendly Cities and Communities (AFCC) agenda, key features of age-friendly communities involve older people, local groups, councils, and businesses working together to improve their communities (25), involving the eight domains of good transportation, good housing, communication and information, outdoor spaces and buildings, opportunities for social participation, respect and social inclusion, civic participation and employment opportunities, and community support and health services. Aligned with the AFCC in the UK, there has been a call to work *with and for* older people to empower them to live independently in the community for as long as possible—enabling healthy and active aging. Specifically, as promoted by the Center for Aging Better (25) and the UK's Industrial Strategy (26), this targets all people to enjoy five extra healthy years of life by 2035. To date, the AFCC agenda has primarily focused on the social and physical environment. Additional elements to address include the psychological sense of place and belonging as well as the needs of seldom heard and under-served populations such as older people living alone, living in rural and remote places and/or living with co-morbidities.

Research indicates that older people in particular struggle with loneliness and social isolation and experience difficulties accessing opportunities for social integration and meaningful participation in a progressively digitized world (27). Spaces and places designed to cater for one population group over another can create schisms across generations and increase the “invisibilisation” of people such as older migrants or homeless youths; thus, normalizing their *absence in “othered” spaces while pathologising their presence in wider society* (28). In seeking to build longer-term sustainable community through intergenerational practices, Kaplan et al. (29) argue for the value of *intergenerational contact zones*, which serve as spatial focal points for different generations to meet, interact, build relationships (*via* trust and friendships), and, if desired, work together to address issues of local importance. Such contact zones can help to enable intergenerational connectivity in spaces and places to shift aspirations beyond creating intergenerational proximity (i.e., “being together”) and toward rich complex social and spatial activities, relationships and places. Wider goals relating to social inclusion and age-integration, participation and design, creativity and cultural understandings, reducing social distance and generating social capital as well as environmental infrastructure can subsequently be pursued together.

It is important to highlight that variations in intergenerational contact zones may be observed across different countries where cultural factors play an important part in conceptualizing intergenerational contact e.g., in collectivist (e.g., China) vs. individualist cultures (e.g., United States). Such cultural differences can shape roles and expectations of younger generations, for example as it pertains to maintaining frequency of social contact and providing care to parents and grandparents.

As part of creating intergenerational spaces and places, research has also identified the importance of environments (i.e., involving the physical, the digital, the trans-regional and social facets of place) that facilitate intergenerational activities to generate greater empowerment, and a strategy for exchanging ideas, enhanced integration and the building of reciprocal and two-way relationships (29–31). Strong intergenerational relationships are not only at the root of healthy and productive aging; they are also an important component of sustainable and livable societies (8). Hence, intergenerational solidarity is an opportunity to create a sense of value, ensuring that people of all ages are an integral part of society (32). Inspired by this principle, the current initiative sought to bring together different generations in purposeful, equitable and participatory co-creation activities to achieve the common goal of developing an intergenerational AFLE.

**Figure 1:**
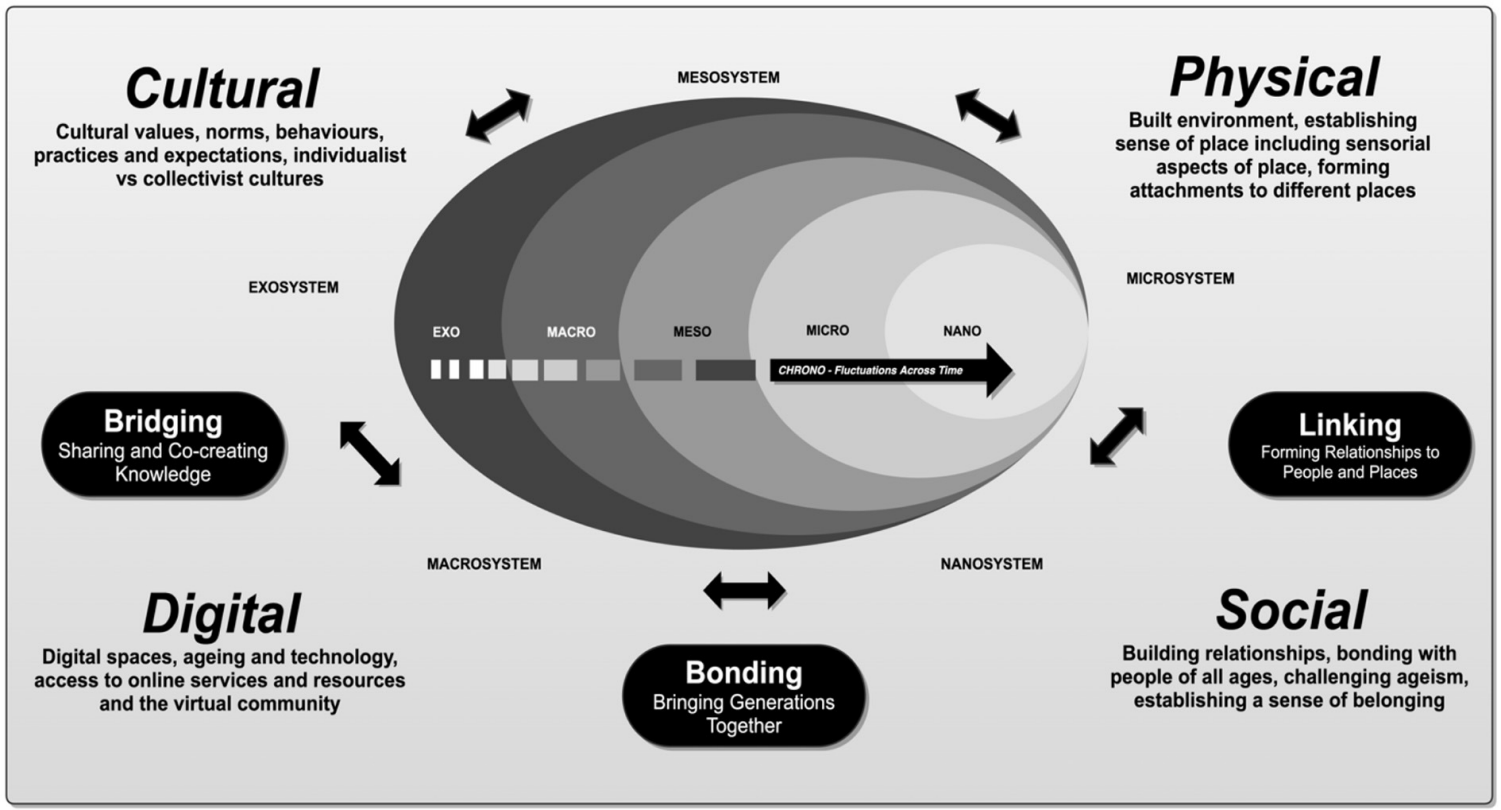
AFLE according to Bronfenbrenner's ecological systems theory. Illustrates how the various ecosystems and levels interact to influence wellbeing.

AFLE is inspired by Bronfenbrenner's ecological systems model and more recently, the socio-ecological model, comprising various systems that interact at multiple levels (33). Figure 1 illustrates how we envision how the various systems and levels interact.

The *nano-system* and *microsystem* combined is viewed as the *individual level* and is the level closest to the individual (i.e., genetics that make up the individual who lives, works and plays in the community, their personal characteristics and aspects of their identity). The microsystem constitutes the immediate environment with which the person interacts. The *mesosystem or inter-relational level* provides the connection between the different structures of the microsystem, for example how local services and amenities support environment, bonding, social interaction, relationship building in physical places and virtual spaces. The *exosystem or organizational or community level* encompasses agencies within the wider social system which impacts people through the mesosystem and microsystems that shape opportunities for social and civic participation (e.g., community centers, cafés, leisure centers). The outermost level, the *macrosystem or societal level*, comprises societal elements such as the prevailing media sources, government, health and social care authorities, technology conglomerates including professional associations which shape societal culture and values, customs, policies and laws. Besides the four levels, the chronosystem adds a temporal dimension, for example, the changes that occur with service transformation or with biological, psychological or social aspects of the adult lifecycle as it pertains to aging. Additionally, a temporal lens enables adaption to change in community and service provisions and in working practices, for example, as was seen in some sectors in response to the pandemic.

For this project, the notion of a “conceptual age friendly living ecosystem” is also applied, and is defined by “a holistic, multi-disciplinary and multi-modular” provision of intergenerational and age-friendly physical, virtual, social and trans-regional places and spaces, with “emphasis on engaging many people in collaborative activities required to move beyond the traditional single cause-effect linear trajectory of a mono-discipline and mono-modal approach” (34).

### 2.2. Study design

CBPR principles were used to guide the design of this participatory co-creation project—co-designed by a mix of academic and non-academic stakeholders from various disciplines (health sciences, urban studies, psychology, architecture and design), age cohorts (5 – 80+) and life experiences in experiential, professional and practitioner domains. This study thus applies triangulation (35) as a technique to enhance the integrity of the findings by, first, bringing together researchers and community-based stakeholders across diverse disciplines and sectors to co-design the study, collect and co-analyse the data; and second, applying multiple methods (e.g., co-creation workshops and qualitative survey) to generate a varied dataset. Both investigator triangulation and methodological triangulation were used to enable multiple explanations of the phenomenon of interest from different vantage points (35).

**Figure 2:**
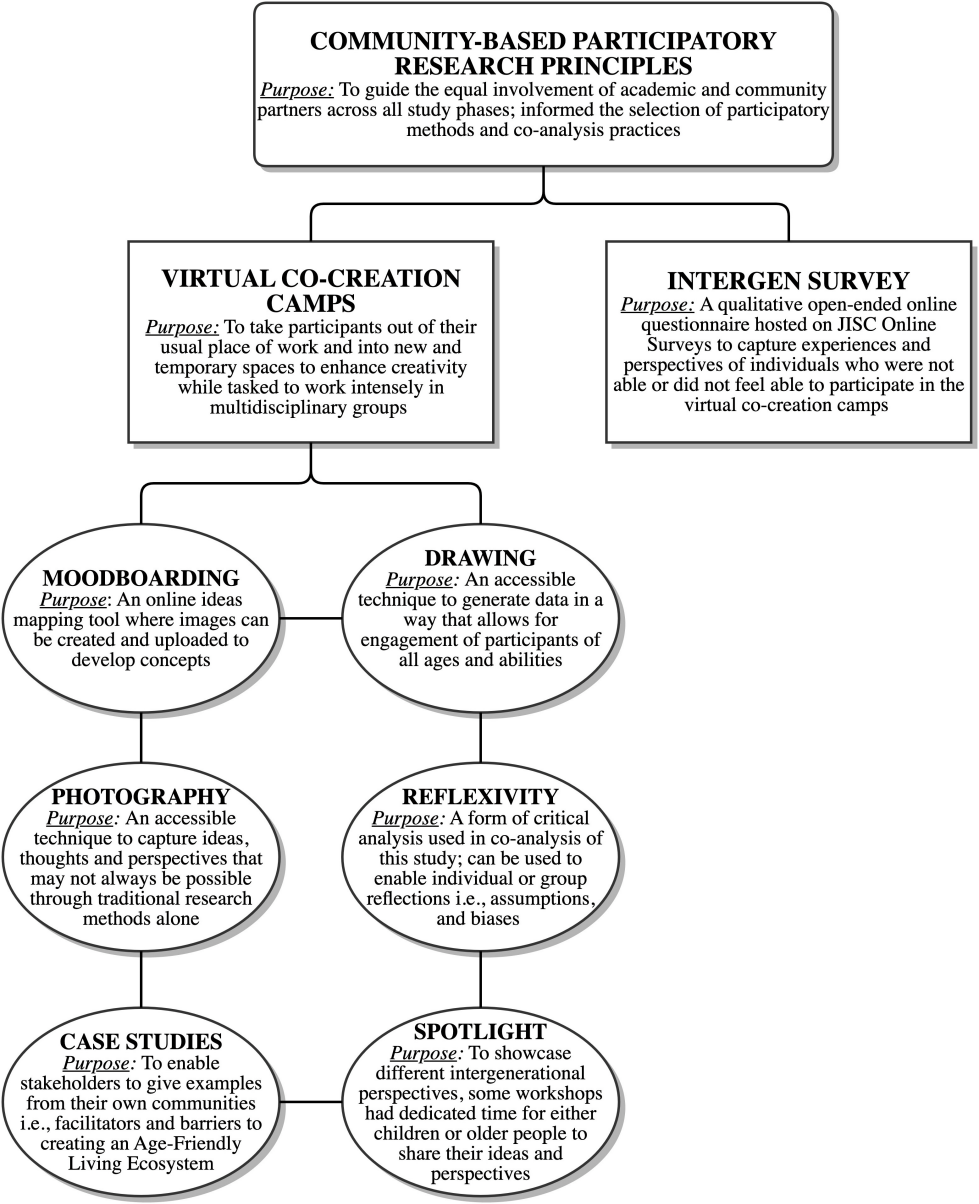
A schematic of the study methodology that includes key research principles which guided the overall participatory nature of the study and the methods used to co-create knowledges and ideas.

Guided by the aforementioned participatory underpinnings and triangulation, multiple methods were used to address project aims, sub-aims and objectives. Designed as in-person engagement workshops, six co-creation camps were planned to facilitate joint-working across diverse stakeholder groups. The idea of co-creation camps stems from the “camp model” of creative-working whereby participants are taken out of their usual place of working and placed in new and temporary spaces to enhance creativity while tasked to work intensely in multidisciplinary groups toward generating innovative ideas, concepts and solutions (36). Due to the COVID-19 pandemic, co-creation camp activities were redesigned to facilitate remote joint-working. Each virtual co-creation camp (VCC) applied digital applications (Zoom, Moodboard) and creative methods (drawing, photography, storytelling, spotlight sessions and case studies) to promote inclusive, creative working between academic and non-academic participants between ages 5 – 80+ years, across eight countries. A brief, open-ended INTERGEN survey was co-designed by the AFLE team to generate ideas for building intergenerational age-friendly places. The survey was circulated widely to capture the perspectives of those who could not participate in the VCCs. A schematic of the methodology can be found in Figure 2.

### 2.3. Recruitment and participants

International participants from eight select countries were invited to join the co-creation study because of their unique insight and expertise for developing inclusive intergenerational spaces and places as indicated in Table 1.

**Table 1:** Indicates broad social characteristics of VCC and survey participants.

	**VCC** ** (*N* = 62)**	**^b^~%**	**INTERGEN** ** (*N* = 130)**	**^b^~%**
^a^ **Age range**				
5–70+	62	100	–	–
13–17	–	–	1	1
18–24	–	–	5	4
25–34	–	–	15	12
35–44	–	–	19	15
45–54	–	–	24	19
55–64	–	–	31	24
65–74	–	–	30	24
75–84	–	–	5	4
**Gender**				
Female	45	73	37	29
Male	17	27	92	71
Prefer Not to Say	–	–	1	1
**Country**				
Australia	2	3	8	6
Canada	3	5	10	8
China	5	9	23	18
Denmark	2	3	7	5
India	3	5	13	10
Singapore	3	5	11	8
Slovakia	3	5	10	8
United Kingdom	41	66	48	37
**Discipline**				
Health related studies	3	5	30	23
Urban studies	2	3	12	9
Housing studies	2	3	4	3
Architecture	3	5	12	9
Materials design	3	5	23	18
Rehabilitation sciences	3	5	3	2
Gerontology	2	3	31	24
Social work	2	3	13	10
Not applicable/prefer not to say	42	69	2	2
^c^ **Sector**				
Academic	20	33	–	–
Industry	8	13	–	–
Health	6	10	–	–
Non-government organizations	17	28	–	–
Younger community members	10	16	–	–

a VCC participants were not asked to specify their age and as such age range (5 – 70+) is provided.

b Percentages were rounded to the whole number.

c Survey participants were not asked to indicate their sectoral affiliations.

A total of 62 individuals from various disciplines and sectors, generations and cultures participated across the six VCCs and 130 participants responded to the INTERGEN survey. VCC and survey participants consisted of multi-disciplinary, multi-sectoral individuals from a range of age groups; but largely constituted female, adults over the age of 24 and under the age of 75. The majority of VCC participants were affiliated with the academic and NGO sectors consisting of older and younger community participants.

VCC and INTERGEN survey participants consisted of a convenience sample of individuals who were interested in the topic area and were willing to participate. Participants were recruited initially using our known networks and subsequently enhanced by a snowball sampling method through electronic circulation of a digital e-invitation across the INN and *via* “word of mouth” through existing national and international academic (e.g., University of Stirling, Heriot-Watt University, Beijing Forestry University, Simon Fraser University, Technical University of Svolen), industry (e.g., StudioPneuma, Architecture and Design Scotland), health (e.g., National Health Service) and non-government organization (e.g., Citadel Youth Center, Children in Scotland) partners who are part of the broader project team. To ensure that the participant group varied in age and was “multi-disciplinary and multi-modular” and aligned with tenets of the conceptual ecosystem model (34), recruitment targeted individuals with lived experience of growing older in community settings from across the lifespan and individuals from organizations with vested interest for creating intergenerational places and spaces.

Participants were asked to provide some demographic information (*via* registration for the VCC event and as part of the INTERGEN survey) that were pertinent to the AFLE co-creation project and for two key knowledge generation activities.

### 2.4. Data generation

Data generation was framed and organized as knowledge co-creation activities in the shape of VCCs and an open-ended qualitative survey (INTERGEN). VCCs were held monthly between May–December 2020 (excluding the summer period). Each session was 2.5 h in duration and were video and audio-recorded in Zoom version 5.3.1 and subsequently transcribed verbatim by the service, TP Transcription. A range of virtual engagement methods were used to enable participation from stakeholders with various skills, abilities, expertise and backgrounds (Table 1). A VCC session agenda was co-developed by the lead author and with input from key members of the project team (JS, AHP, PS, RP, and RW). Details of the session including the objective, activities, outcomes and outputs can be found in Table 2.

**Table 2:** Key objectives, activities and the resulting outcomes and outputs of the VCCs.

	**Objective**	**Activities**	**^a^Outcome and Output**
VCC1	▪ To define features of an AFLE	▪ Technical tutorial; brief presentation; intergenerational case study (Healthy Universities for Healthy Communities); visual ideas mapping using online Moodboard; spotlight on children's perspectives; breakout groups exploring AFLE; group discussion of interpretation under COVID 19	▪Outcome: Determined features of an AFLE for VCC2 ▪Output: a conceptual map of ideas (Supplementary Figure 1)
VCC2	▪ To explore the first steps toward co-creating an AFLE	▪ Reflections from VCC1; intergenerational case study (Developing Intergenerational Places in Dumfries, Scotland); spotlight on older peoples' perspectives; breakout groups exploring AFLE steps (people & place, culture & relationships, connection and learning); full group discussion of ideas)	▪Outcome: Determined ideas for aim, objectives and important questions for an upscaled AFLE co-creation project
VCC3	▪ To co-analyse VCC1 & 2 (AFLE team only) data to refine VCC2 outcomes	▪ Presentation overview of data and ideas for an aim, objectives and important questions; full group discussion; working session to refine aim objectives, important questions and developing a conceptual model	▪Output: Aim, objectives, important questions and a conceptual ecosystem model (Supplementary Figure 2) for an upscaled AFLE project
VCC4	▪ To determine what is needed to develop AFLE	▪ Presentation of aim, objectives and important questions and conceptual model to AFLE consortium; intergenerational case study (Intergenerational Place-making in Canada); breakout groups (develop community of practice; capture diverse voices; sharing what we learned)	▪Outcome: Determined ideas necessary activities to develop AFLE
VCC5	▪ To explore opportunities for inter-generational policy and practice	▪ Overview of current state of intergenerational policy; intergenerational case study and spotlight on planners (Policy & Planning Initiatives in Scotland); discussion and co-creation of a policy road map	▪Output: Road map for policies toward an intergenerational AFLE (Supplementary Figure 3)
VCC6	▪ To explore opportunities for knowledge translation (KT) and impact for AFLE	▪ Intergenerational case studies overview; breakout groups (opportunities for intergenerational KT and impact); spotlight on young people *via* a KT youth presentation (Challenges of Intergenerational Communication); full group discussion on next steps	▪Output: Proposal for an upscaled longitudinal study ▪Outcome: Determined activities for pathway toward impact

a The outcome and output of each VCC are noted in this column. The outcome indicates the achievement of each VCC, while, the output is what was created at the end of each VCC.

The VCCs were facilitated by the project co-leads (co-authors: MLF, JS, and AHP) and consisted of a purposeful composition of participants aligned with the VCC objectives. To accomplish each VCC objective required novel knowledge generation activities to facilitate participant engagement across multiple generations. Activities such as the use of case studies, spotlight sessions, storytelling and Moodboard (an online application which compiles images and drawings participants have produced into a collage) helped to enable participation and encouraged rich discussion from children, adults and older adults. Subsequently, the activities generated a range of outcomes and produced innovative visual outputs and rich description (see Supplementary material 1–3). In each VCC, a spotlight session was held which focused on experiences and thoughts from particular population representatives such as children (VCC1), young people (VCC6), older people (VCC2) and planners (VCC5). Importantly, the process of the VCCs was iterative, with each building on the former camp so that the distinct outcome and/or output from each preceding VCC created the foundation for discussion and co-creation for the following session.

The INTERGEN survey was co-developed by the project team (co-authors: MLF, JS, AHP, PS, and RP) as a qualitative open-ended online questionnaire hosted on JISC Online Surveys. The purpose of the INTERGEN survey was to capture experiences and perspectives of individuals who were not able or did not feel able to participate in the VCCs. As such, a power calculation to determine a suitable sample size was not necessary for the purposes of this project. There were no strict inclusion/exclusion criteria for participation. Any individual with an interest in the topic area who was willing and able could participate. The INTERGEN SURVEY covered the following issues: demographic information, everyday multigenerational interactions; intergenerational and age-friendly place features; multigenerational place attractions; multigenerational place safety; and necessary stakeholders required for creating intergenerational and age-friendly places. The INTERGEN survey was launched May 16, 2020, and closed February 15, 2021. Survey questions can be found in Supplementary Table 1.

### 2.5. Data analysis

An experienced qualitative researcher (co-author RR) with input from co-leads (co-authors: MLF, JS, and AHP) conducted a reflexive thematic analysis across both the VCC and INTERGEN survey data to generate themes and patterns based on Braun and Clarkes work (37, 38). The INTERGEN survey results were compiled, and organized using the analyze function in JISC Online Surveys and subsequently exported, reviewed and co-analyzed descriptively by the three project co-leads *via* the Zoom communications platform.

Analysis began with a read-through of each transcript for familiarization with the dataset. An initial coding structure was created by key members of the project team (co-authors: MLF, JS, AHP, RR, PS, and RW), based on low-level/descriptive coding that resulted from coding units of text by labeling with a word or phrase closely related to the participant's account (39). This was conducted initially for each VCC transcript separately. Through an iterative process of reading and rereading the text, codes were subject to interpretation and notes were made to define the relationship between codes on the basis of similar meanings. Codes with similar meanings were combined into larger patterns and structured as initial themes. These initial themes were then further interpreted and defined through team discussion. Any conflictual meanings and interpretations were resolved through discussion. This resulted in a detailed coding structure and framework agreed upon by two additional qualitative team members (co-authors: MLF and JS). Guided by the coding structure and framework (Table 3), a thematic co-analysis of all 6 VCC transcripts was conducted by the three qualitative researchers (co-authors: RR, JS, and MLF) with discussion input from the wider team (co-authors: PS, RP, RW, and RC). Finally, guidelines and recommendations for the co-design of intergenerational spaces and places for older people were generated from the data analysis in a process of deliberative dialogue (40).

**Table 3:** Snapshot of thematic analysis coding structure and framework.

**Theme**	**Code**	**Quotation**
Sensory factors: feeling and emotion as starting points for physical design	Sensorial aspects of place	We started our discussion talking about the design of spaces with the senses in mind and how we all share smell, and taste, and touch, and things like the feel of the wind and different things. (P15)
	Relational vs. sensorial design elements	We talked a little bit about companionship and love and those kinds of more emotional aspects, how do we design those into environments? (P14)
Physical and digital factors: Designing AFLE spaces and places	Focus on universal benefits	…bigger awareness-raising project, to just make everyone is aware of what the benefits of intergenerational work is and are” (P18)
	Apply assets-based community development	I think we need to have a bottom-up approach to understanding what is the existing assets of a place and that could help us inform, like, what do we need in this place and where is the best place to locate things? (P17)
Social and cultural factors: tackling ageism and exclusion as part of the solution	Make visible diversity of older people	…I think we are contributing to ageism when we don't show the other side as well. I know there is a lot of focus on health issues so, you're always looking at older adults as people who are incompetent, or they need support—they need somebody to help. So, we need to show the world that there are older adults who are very involved in their communities and working very hard so, we need to see them as well. (P18)
	Consider heterogenous experiences/needs of older people	So, you know with sheds, they're very much gendered spaces and I think that is something that we need to take into account, that notion of which places feel right for various genders and how do we take that into account? (P24)

Thematic findings reports were produced and circulated prior to each subsequent VCC. Findings were also presented on the day of the session whereby participants amended and/or confirmed the interpretation to ensure accuracy.

### 2.6. Ethics

Consultation with the School of Health Sciences Ethics Convener at the University of Dundee determined that institutional ethical review and approval was not required due to the co-creation rather than research-oriented nature of this project. However, standard code of ethics and conduct was applied according to the British Psychological Society Ethical Standards and Guidelines. As well, all handling of data and data management were complaint with EU General Data Protection Regulation (GDPR). All participants provided informed consent and permission to be video- and audio-recorded; and for the data from both the VCCs and INTERGEN survey to be used for publications and in other widely circulated knowledge sharing materials. “TP Transcription” a transcription service provider verified by the University of Dundee for EU GDPR compliancy was used. Transcripts were de-identified by the transcriptionist to ensure confidentiality. No adult participants were provided compensation for participation. Children and youths from third sector organizations were provided toy gift cards and a pizza party was provided (outside of the COVID lockdown period) as a token of appreciation for their contributions.

## 3. Results

The following results and subsequent discussion of the findings were developed in the research and reproduced in the end of project report (41), which can be found in the electronic repositories of the University of Dundee and the Scottish University Insight Institute. The findings represent both the reflexive thematic analysis of Virtual Co-creation Camps (VCCs) and the INTERGEN survey results. As survey questions were mostly open-ended, the findings from the survey are therefore presented as qualitative findings. Where yes/no answered were requested, basic descriptive statistics are given. The integrated survey and VCC findings are addressed in three main themes that highlight the necessary characteristics of AFLEs as suggested by the VCC participants and survey respondents. These are: (i) Sensory factors; (ii) Physical and digital factors; and (iii) Social and cultural factors. Where particular participant quotes or thoughts are identified within the findings below, they are referred to by their participant number allocated during the anonymization and transcription process. In this way, VCC2, P4 refers to participant number 4 in the second VCC workshop held.

### 3.1. Sensory factors: Feeling and emotion as starting points for physical design

Through the INTERGEN survey, sensory qualities of intergenerational places were often identified from the perspectives of older people's (or their carers') needs; for example, noise control was important for people living with hearing impairment and people with autism with sound sensitivities. Lighting was seen as important for lip reading. Other considered sensory aspects of intergenerational places were based on activity, e.g., the sounds and sight of children laughing; a baby crying; smells from food cooking; people cycling; people using smart phones; athletes playing sports; and people playing music. Sensory aspects, if well designed into place, were felt to bring a sense of warmth and friendliness thus making the space more inviting. Indeed, some survey respondents gave more general responses such as places and spaces needing to be comfortable, “generally welcoming for all ages,” friendly.

According to the INTERGEN survey participants, indicators of good quality intergenerational age-friendly outdoor spaces that enhanced sensorial aspects of place were connected to physical design. These included: “weatherproof areas”; “toilets accessible to all”; “well-lit sidewalks that are in good shape”; “inclusive public seating”; “green space”; “access to public transportation”; “art and play structures”; “openness for sports”; “having skateboard friendly slopes”; “safe buffer zone between pedestrians and traffic”; “walking and cycle friendly sidewalks”; “clear sightlines and signage”; “benches, tables, and seating at different heights”; “no trip hazards”; “allotments for community gardening”; and, overall, spaces that are “safe and secure, non-threatening, and welcoming” to all people.

Relatedly, in VCC1, P3 noted that “*sensory stuff is really important in terms of accessibility”* and experience of place, thus consideration of varying sensory needs could be crucial for promoting AFLE inclusivity; for example, noise level might need to be a priority for engaging people affected by dementia and autism while lighting can be critical both for fading vision but also creating comfortable atmospheres. While the conceptualization of an AFLE needs to be focused and attainable, designing for support, opportunity, meaning and sensory experience is critical if diverse needs are to be met and multi-level constraints countered. The discussions in VCCs on inclusivity helped to reveal the importance of “centring in the margins” (42), in prioritizing those who are living outside the margins of society, and those with the most to gain from intergenerational initiatives.

Sensory and emotional needs should be considered as starting points for design. These foci can connect people and contribute to wellbeing for all ages: “*We started our discussion talking about the design of spaces with the senses in mind and how we all share smell, and taste, and touch, and things like the feel of the wind and different things.”* (VCC1, P15)

Design guided by sensory experience needs to be inclusive of peoples' varying ability to use their senses, particularly due to age and disability. One participant (VCC3, P6) raised the importance of considering acoustics in a place to ensure connection is possible through speaking and listening. In addition, design that facilitates relationship-building was deemed particularly vital for meeting emotional needs: “*We talked a little bit about companionship and love and those kinds of more emotional aspects, how do we design those into environments?”* (VCC1, P14)

It was acknowledged that it can be difficult to describe emotional experiences of a place, and thus challenging to apply to design. Yet, there was agreement that emotional experiences are important to consider, such as a sense of belonging: “*If we think we belong there, we're more likely to use it, we're more likely to enjoy it.”* (VCC2, P14)

Meeting sensory and emotional needs through design could also be achieved through opportunities created to connect people of all ages with the outside environment. This was deemed particularly important given the increased use of technology to facilitate connection, especially given the circumstances of COVID-19 (7).

Participants strongly emphasized the importance of the emotional and sensorial experience of a place, particularly to facilitate a sense of shared intergenerational humanity.

### 3.2. Physical and digital factors: Designing AFLE spaces and places

Turning again back to the INTERGEN survey findings, when participants were asked if they had ever been to a place that they considered “intergenerational”, the majority (*n* = 75: 84%) reported “yes”. When asked to indicate what constitutes features of an intergenerational and age-friendly place, three categories of features were reported: physical and virtual environmental aspects were mentioned as important for optimizing health and wellbeing outcomes for people in places and across the lifespan. Physical features were defined by participants as mainly comprising built environments that enabled accessible and flexible indoor and outdoor spaces for people of all ages.

Characteristics of good indoor spaces for all age groups were highlighted as: “colorful”; “secure and friendly”; “not too cold”; “multi-use and multi-purpose”; “accommodating mobility aids and people with mobility issues”; “bright with good lighting”; “well-spaced to accommodate for social distancing”; “having artwork that enabled a sense of shared ownership”; “having good signage with pictures and words”; “having sound damping”; “having good seating”; “high quality toilets”; “having automatic door entrances and elevators”; “having color contrast between floor and walls”; “having secluded corners for peace and privacy”; “having non-slip floor”; “having different heights of tables and chairs”; “having light switches and electric sockets at the correct height”; “having views to outdoors”; “having plants”; and “generally welcoming for all ages”.

In terms of creating inclusive and intergenerational environments, VCC participants highlighted that the aim and impact of the design of space and shared activities should not only focus on the notion of age, but rather the emphasis should be placed on “*bringing the community together”* (P13). The design of the physical environment and opportunities for shared activities was emphasized as an imperative driven by the community. During VCC1, P12 shared an example from the Singaporean context whereby the location of schoolchildren and people living in a care home were connected with commercial shopping areas to facilitate intergenerational interactions. Diverse community stakeholder engagement was deemed important not only for developing the physical and structural design changes on a community level, but also enabled the co-creation of solutions to adapt spaces in contexts where “custom-built” (VCC2, P6) environments were not feasible.

VCC participants further identified the importance of normalizing community investment in designing physical intergenerational and age-friendly spaces and places. One participant emphasized the need for “*commitment to intergenerational spaces and this way of working together intergenerationally so that we're not always chasing funding, and so we have a commitment to this being the norm”* (VCC6, P6). It was felt that embedded normalization can help to ensure sustainable development through sustained funding.

During the VCC discussions, several queries were raised related to how age-friendliness can be embedded into the design of spaces and the funding bodies that support building projects. For instance, one participant questioned “*how we make it [an intergenerational age-friendly environment] so that it becomes part of what communities do rather than just something that occurs when the money is there”* (VCC5, P1).

There was a sense that investing in physical design and adapting spaces toward intergenerational or multigenerational age-friendliness can be cost-effective. Adapting existing places to be useful to people of all ages can be more cost-effective than “acquiring land and building from new” (VCC4, P2). As well, creating multigenerational spaces could benefit people not only as they age but also as the contexts in which they live change. As VCC5, P1 explained, public health and urban planners should consider “*opportunities for people as they age, but also in response to changing environments.”* The focus on the dynamic and fluid aspects of place emphasizes that such infrastructural developments would be relevant to people not only as they age but also as they adapt to changes in their circumstances. Overall, there was consensus that the universal benefits of multigenerational use of spaces over time outweigh the benefits of focusing on creating spaces and places for only “older” or “younger” people.

Meanwhile, technology was identified as playing a potential role in supporting the development of virtual intergenerational spaces that can include a community of practice of younger and older people to further the intergenerational AFLE agenda. In particular, the circumstances of COVID-19 required considerations of virtual means of engagement and access to vital health and social care services and resources for all people. For the AFLE project, video-conferencing and design technology, and its potential for initiating and maintaining relationships was highlighted.

VCC3, P4 noted that technology should be seen as the facilitator of activity and not the main outcome: “*We talked a bit about hackathons… and not just about coming up with technology-based solutions but about them being the beginnings of a relationship that would grow from there.”* Even so, caution was raised in relation to relying too heavily on technology given barriers to digital access and culturally based negative perceptions of technology: “*In some cultures, technology is seen as a threat by older people, so we need to be wary of that and not just go down that technological route”* (VCC3, P4). Nevertheless, the need for technology alongside traditional methods of engagement was identified as important for reaching diverse participants: “*we include everybody, and it doesn't always have to be the technological side of things”* (VCC3, P7). Careful consideration of the intended and unintended consequences of technology are suggested, as technology was viewed as creating opportunities but also for their potential to exclude groups from access to services and supports.

All in all, as emphasized in both the survey and VCC findings, there are a range of dynamic considerations to be made when co-designing physical places and virtual spaces. Both are in some way interconnected and have intended and unintended positive and negative consequences that must be acknowledge for the development of intergenerational AFLEs.

### 3.3. Social and cultural factors: Tackling ageism and exclusion as part of the solution

Aside from sensory, physical and digital features, the INTERGEN survey respondents reported the need for socio-cultural design considerations, aligned with a social justice perspective. For example, there is a fundamental need to ensure that housing, transportation and activities of daily living are affordable, well-designed for accessibility, safe and inclusive for all people across generations. It was stressed that any member of the community, regardless of age will have something to offer, but to enable, as Heu et al. have pointed out (43), collectivism vs. individualism, pervasive ageism and stigma must be addressed so that AFLE initiatives build welcoming, safe and inclusive places and spaces for people of all ages and cultures.

Survey participants were asked to indicate the frequency with which they interacted with people who were young or older than themselves, outside the immediate family, in their everyday lives. When asked, “in normal circumstances, how often do you interact with people who are younger than you (outside the immediate family)”, over half the participants (*n*=75; ~58%) reported “often”, a third of participants (*n* = 39; ~30%) reported “sometimes” and one in ten participants (*n* = 14; ~11%) reported “seldom”. No participants indicated having rarely interacted with individuals younger than they were on a day-to-day basis. Similarly, when participants were asked, “in normal circumstances, how often do you interact with people who are older than you (outside the immediate family)”, over half the participants (*n* = 79; ~61%) reported “often”, a third of participants (*n* = 43; ~33%) and only 6% of participants (*n* = 8) reported “seldom”. None of the participants had indicated having never interacted with individuals older than them on a day-to-day basis–although interactions with older people were reported to be slightly more frequent.

Accordingly, the challenges of reducing social distance and bringing the diversity of people together regardless of age, gender, ability, sexuality, religion and culture requires acknowledgment, acceptance and appreciation for the unique differences of individuals and groups.

During the VCCs, the complexity of designing an AFLE and building into it opportunities and supports to bring multigenerational groups together was identified. For example, in the design, development and planning stages, effective engagement of all relevant stakeholders, including children and older people was highlighted. A ground up approach to engagement was felt necessary, beginning with all stakeholders discussing and working toward transformation of negative to positive ageist stereotypes, for instance by “*bigging up our older people who are thriving”* (VCC5, P11), and making ageism visible so that it can be challenged:

…*I think we are contributing to ageism when we don't show the other side as well. I know there is a lot of focus on health issues so, you're always looking at older adults as people who are incompetent, or they need support—they need somebody to help. So, we need to show the world that there are older adults who are very involved in their communities and working very hard so, we need to see them as well*. (VCC5, P18)

*We don't want the young people only to know older adults as those who are frail. We also need to find ways to make sure we bring ‘the well' older adults, who live in your community and how can we bring them together*. (VCC6, P20)

Emphasizing the heterogeneity of people rather than a focus on just age was seen as a step forward in designing AFLEs, as one participant explained:

*Sometimes we will ignore their age because we think their physical condition is more important because if they can live by themselves or they need to use a wheelchair or they need others' help… that will make a big change about what kind of furniture they will use*. (VCC6, P9)

Assumptions made about individuals' experiences based on old age was felt to limit possibilities of engaging with them effectively. The universality of ageism was seen as a threat to AFLE conceptualization, involvement and design as P8 pointed out, to have universal appeal “*what we're talking about affects everybody*” (VCC1, P3. The prevalence of internal ageism (whereby older people constrained themselves because they are older) was a further constraint which made strategies to enhance their visibility more challenging.

The focus within AFLE on diversifying engagement highlighted barriers to participation in designing and living in age-friendly communities. Ensuring AFLE designs were sensitive issues of marginalization and intersecting social characteristics (such as socioeconomic status, sexual orientation, and race/ethnicity) were discussed and strategies to effectively address this were raised, mostly based on provision of active, knowledgeable support in community and service settings:

“*It's…more than just opening the door, you've got to help people through the door as well… it's about going, “Well, this is what we're doing, and we would really like you to come, and we'll help you get there.”* (VCC1, P21)

In terms of potential solutions, the diversity of national networks, community hubs and international participants involved in the AFLE project provided an opportunity to discuss the ways in which we can tackle issues of inclusion in the community:

*...the kind of context is everything though and so some of those hubs—the hub in India—the experience of being an LGBTI person in India is very different to being someone who identifies in that way in Scotland or in Canada or wherever else. And so, I think there is something about encouraging everyone to engage through those networks..*. (VCC4, P21)

*When we've spoken to people about who it is in their community, they want to know it's not defined just by being a teenager or being over the age of seventy; it's connecting with young mothers, it's connecting in the middle as well*. (VCC4, P21)

For example, age-friendly networks of community champions with the knowledge and skills to “*help people through the door”* (VCC4, P21) were deemed useful in overcoming participatory barriers. In addition to identifying the value of strategising on how to increase the diversity of creators and community members of age-friendly living environments, the importance of reaching people who might benefit most from AFLE benefits was acknowledged, by building on “*the people who are already quite well connected, are already doing okay”* (VCC4, P23). Forging new connections on the basis of commonalities, shared needs and extant knowledge (rather than based on age) was another inclusion strategy in designing and participating in age-friendly ecosystems:

…*thinking about pensioner's associations, youth groups, housing associations—schools, and having clubs in schools where it's not just about learning—that's where people make real connections and there are lots of other things going on there; that's where life happens*. (VCC3, P12)

…*not just being disseminated from one age group or one particular person in society but actually being shared to and by the whole community together and lots of ideas about buddy walks and YouTube videos, those kinds of things*. (VCC2, P1)

Akin to the notion of heterogeneity amongst older people, providing opportunity to enhance critical thinking into different social identities, positionalities and issues was seen as crucial to addressing the complexity of AFLE conceptualization and design. For example, thinking about how to include sensory elements, experience and meaning into AFLE involves, not just functionality and accessibility of physical space but also psycho-social considerations such as confidence and belonging, gender roles and expectations, and resources like information, money, and transportation.

…*we know that age doesn't cover all of the different aspects of a person when they are trying to use, to access, to develop meaning with space. And so, I think, disability, we've talked about gender, and we've talked about age. So, maybe there needs to be some kind of recognition of intersectionality*. (VCC3, P2)

…*as I am trying to take a baby out in the pram, and things and as I'm trying to access spaces, many of these spaces are not really accessible to us as well. So, that is how it can be something that is much more across all the age groups*. (VCC3, P6)

A question linked to those themes pertain to how “*longevity*” (VCC3, P6) of intergenerational spaces as well as the activities enabled by these spaces can be ensured. The imperative to gain community-level resources and funding investment was subsequently extrapolated to concerns about climate change, and the potential ecological benefit of investing in age-friendly living spaces. One recommendation focussed on the ways in which people who could champion the development of intergenerational spaces can be identified and supported. This was viewed as crucial for achieving sustainable age-friendly living environments.

Involving local people of all ages at a national and local level and from a wide range of sectors from the beginning of a design project was highlighted by participants as essential for implementing an AFLE in practice. Broadly, this strategy was identified as particularly useful for helping to overcome resistance to change in a community and among potential funders. The initiation of policy planning and practice from the ground-up, community level was deemed essential because, “*it is really, really vital if we get the right kind of places that people want; not what architects want or planners want or transport engineers want, but what people want and need*” (VCC2, P16). It was suggested that people of varied ages can have common wants and needs (e.g., walking paths) and there was an emphasis on the importance of accessing, and having the appropriate mechanisms for amplifying the voices of all ages to inform community-led policies and practices for age-friendly environments:

…*how we get those different voices to be heard to create those better environments. Whether they be urban environments, rural environments, whether the physical spaces, the interior spaces—how do we capture those intergenerational needs from these different voices and how can we actually apply that to a policy to make a change to improve these spaces and places, to make them more accessible*. (VCC4, P10)

Organizing communities around key concepts such as ‘universal accessibility' was deemed a strategy for planning policy and practice. A potential focus for engaging community members in policy and practice discussion was to undertake awareness raising about the topic. One participant suggested a “*bigger awareness-raising project, to just make everyone is aware of what the benefit of intergenerational work is and are”* (VCC1, P18). This awareness-raising strategy aligns with the goal identified to mainstream age-related issues into policy and practice rather than, for example, develop specific policies for older people. Appropriate messaging about intergenerational policy and practice could engage people who do not realize the relevance of the topic to them in discussions as well as supporting policy mainstreaming of age-related issues.

## 4. Discussion

Constant change, including those arising from technical developments, extends possibilities for rich social interaction for some, yet has potential to hinder long-established means of interacting for others. To address this challenge requires a knowledge pathway toward a better understanding of how multigenerational people can better interact with fluctuating social domains (industry, voluntary sector, public service, transportation, academic/university) to facilitate mutual social engagement. Findings from the AFLE project have highlighted innovative and promising concepts and ideas in the shape of co-produced outputs (see Supplementary Figures 1–3).

Reflexive team-based thematic analysis of VCC discussions indicate that intergenerational and age-friendly environments can benefit people of all ages, and positively impact not only at the micro-level (i.e., individual) but also at the meso- (i.e., families, communities), macro- (i.e., cultures, societies), exo-levels (i.e., institutions, industries) shaped by paradigmatic socio-cultural changes across time. The potential for design to facilitate and embed opportunities for shared experience across generations is for better wellbeing of society.

Aligned with VCC findings, INTERGEN survey results highlighted barriers and facilitators to implementation of intergenerational and age-friendly environments, particularly as it pertained to designing and modifying indoor and outdoor places and virtual spaces. The analysis revealed that intergenerationality and inclusivity of space can be promoted *via* individual (micro) levels including sensory (e.g., sound damping, lighting, greenery), physical (e.g., non-slip floors, signage), virtual (e.g., optimizing the role of technology), social (e.g., challenging negative discourses about younger and older people) and cultural [e.g., promoting collective responsibilitisation (44)] modifications, enabling inclusive access to shared spaces regardless of age, ability and cultural background. Intersectional considerations relevant to other personal characteristics such as socio-economic status, religion, and race/ethnicity would be worth examining. Making better use of the natural environment, particularly during the COVID-19 lockdown, was also highlighted. Further exploration of potential designs and uses of the natural environment for enhancing age-friendly environments, including concerns about climate change and weather, is a priority.

Regarding macro- and exo-level concepts and ideas, it is important to note that at its core AFLE is informed by existing policy. For example, in the UK, the focus on intergenerational communities builds upon policy which has sought to deliver an intergenerational approach including the “Society for All Ages” moving from a focus not just on “older people” toward a more holistic perspective for social policy aimed at all generations (45). On a global front, the aim of AFLE sought to align with the United Nations (UN) Sustainable Development Goals (SDGs) focussing on “mobilizing efforts to end all forms of poverty, fighting inequalities and tackling climate change, while ensuring that no one is left behind” (16). The innovative conceptualization of AFLE in this project is linked to progressing SDGs 3 (to “ensure healthy lives, promote wellbeing for all at all ages”) and 11 (making “cities and human settlements inclusive, safe, resilient and sustainable”), through incorporating experiences and expertise from stakeholders across 8 countries that enabled different cultural perspectives to emerge.

To advance intergenerational policy and practice, VCC participants emphasized that this needs to occur from the ground-up with relationship building involving diverse intergenerational community representatives who can offer insights into the everyday realities of older and younger people. Strategies identified to achieve effective community-level engagement include organizing activities that are fun and meet wellbeing and sensorial needs, ensuring power balances are maintained across professional, practitioner, academic and experiential stakeholders as well as bringing people together to talk about topics that are universally relevant, concrete in nature and awareness-raising. Cultivating trust and confidence among community members and professionals in the process of policy and practice development and implementation is essential—facilitated by an ethos of valuing involvement, understanding community, investing in wellbeing, and sharing stories of everyday lives.

### 4.1. Directions for public health and urban planning

Findings from this project present important implications for public health and urban planning when cultivating inclusive and age-friendly environments. An age-friendly environment involves a physical design that embeds and facilitates opportunities for people of different ages to connect on a regular basis through shared purpose and experiences and to develop cross generational relationships of mutual benefit. This can be achieved by addressing needs, interests and leisure. It implicates in the design of intergenerational places, the need for attention to the sensory experience of place, the way in which socio-physical environments promote or generate feelings and emotions (bringing ambient environment into focus), sense of safety and belonging, and enjoyment of activities such as eating, playing, and learning.

Space and intergenerational relationships and programming that are enabled by meaningful places co-created in participatory ways need to be designed for sustainability so that they remain useful and accessible over time to diverse people of all ages. Analysis VCC and INTERGEN survey highlighted that in order to support intergenerational engagement activities for public health programming should, at minimum, consider the role of technology, the heterogeneity of older people, barriers to engagement on the basis of characteristics intersectional with age, the role of relationships, and the role of place. Furthermore, the analysis also revealed that designing with physical and emotional safety in mind underscored the need to design out environmental hazards such as trip hazards and with a need to focus on both family-based interventions as well as for the requirements and enjoyment of young people.

Regarding universal design, while the merits of universal design (particularly in interior places) was commended for promoting the independence of younger and older people and making life easier for people of all ages, the difficulties of producing effective, vibrant, well used universal design in a diverse, inclusive, and accessible environment were recognized. Here, universal design was envisioned as a design process that empowers a diverse population to improve their performance, health and wellness, and social participation by creating conditions which target sensory, physical, virtual, social and cultural aspects of place that work jointly to shape human agency (46). This evades some of the critiques of universal design as providing environmental or technical fixes for singular problems and emerging from professional perspectives rather than being co-produced with the target population. However, care needs to be taken in intergeneration design for older people, that usability and accessibility are not prioritized over the socio-psychological- or cultural meanings associated with spaces and places.

Universal design, designing for multiple sensory, experiential, leisure, and functional spaces, interconnecting across community assets all require sustained financial backing, engagement, and action on the part of community members and commitment from local and national government, commerce, and industry. Without this, progressing from the siloing of generations through service provision, especially young people and older people, in spaces and places, as found by Cushing and van Vliet (30), is likely to continue and the normalization of everyday intergenerational living is less likely to naturally emerge. As Kaplan et al. have argued (8), intergenerational societies need to embody both sustainability and liveability.

Meanwhile, an assets-based approach in designing links between people and community services was also highlighted in the workshops, suggesting that age-friendly development is not only about physical infrastructure but should also consider mapping the resources, facilities, people, places, and services that use a community area or virtual spaces and how best to identify and integrate across them. Subsequently, this would generate a network of interconnected community assets that link together in supportive structures, substantially reflecting the socio-ecosystem approach to community participation. Embedding the notion of a socio-ecological system in public health initiatives should involve the individual person, their relationships, local communities, and organizations (health and social care, voluntary and community organizations, leisure, retail and private and public businesses) working together to provide the inter-related contexts for sustainable support and liveability.

Regarding public policy recommendations, findings indicate that the development of intergenerational policy and practice must involve ground-up community-based conceptualization, relationship building, and involve diverse intergenerational community representatives. Strategies identified to achieve effective community-level engagement include organizing activities that are fun and meet wellbeing and sensory needs, ensuring power balances are maintained across professional, practitioner, academic, and experiential stakeholders. This is in addition to bringing people together to talk about topics that are universally relevant, concrete in nature, and awareness-raising and can be done through events and messaging focussed on facilitating genuine engagement (47, 48); and accelerating progress with mainstreaming age-related policies and practices.

Cultivating trust and confidence among community members and professionals involved in the process of policy and practice development and implementation is essential. Trust and confidence are needed for creating relationships among diverse community members and professionals as well as ensuring that policy and practice development and implementation processes are deemed fair and representative. As emphasized by participants, this can be facilitated by developing a community of practice and sharing widely what has been learned about intergenerational and age-friendly design.

To realize the strategies identified for ground-up community-led engagement that include intergenerational policy and practice development and implementation, discussion of how to overcome perceived barriers (e.g., time for relationship-building), facilitate opportunities (e.g., messaging, communications, empowerment), and build on current known facilitators is needed. When creating intergenerational policy and practice, avoidance of a “them and us” attitude is crucial. It can be achieved by highlighting and challenging normative power relationships that tend to prioritize professional voices over those expressed by younger and older people and local communities. These strategies can help to inform local urban planning guidelines to ensure age-friendly intergenerationality is threaded throughout all planning practices.

Regarding next steps, the themes signal the importance of producing a public health and urban planning strategy map for progressing intergenerational AFLEs—accessible and fit for purpose across cultural contexts, that highlight the benefits of multi- and intergenerational spaces for changes in both personal and global circumstances, that are inclusive in terms of language and cultural differences and are considerate of intersectional social identities. Identification of key beneficiaries and a thorough and robust evaluation of public health planning, research and urban design processes was also recommended as an instrumental next step for ensuring the development of sustainable and liveable intergenerational environments.

Last, interest in multigenerational housing for intergenerational living was perceived as a major step toward development of Intergenerationality for age-friendly development. Interest in co-housing may be increasing due to COVID-19 whereby communities are encouraged to live in multigenerational housing and to come together to support those in need (7). However, knowledge on how to *do* this effectively needs to be further developed. Consequently, this was identified as an area for future research.

### 4.2. Strengths and limitations

The success of the AFLE project—as a co-creation initiative implemented at the height of COVID-19 was the product of novel design and use of unorthodox and multi-methods approaches. First, AFLE was underpinned by participatory principles of equity, empowerment, inclusion, and partnership and operates against oppressive practices to ensure reciprocal transfer of knowledge and expertise; inclusive participation; power sharing and equity; and knowledge ownership across the AFLE consortium, which included an international group of participants from multiple generations, cultures and geographies (49). Second, AFLE used bidirectional methods of knowledge development, exchange and translation (e.g., moodboarding, drawing, photography, reflexivity, case studies and spotlight sessions) to engage knowledge users throughout the entire cocreation process. This began with the identification of an aim, objectives and important questions, followed by the knowledge generation activities, and pathways toward generating and sustaining impact to develop intergenerational and age-friendly places and spaces. Using atypical methods was grounded in the assertion that “researchers and knowledge users are both experts bringing complementary knowledge and skills to the team” and “collaborate on issue-driven research with the expectation the research will generate implementable solutions to long-standing problems” (50).

Despite notable strengths, for the VCCs, a key limitation was the digital nature of the engagement process. Some invited participants chose not to participate using the Zoom platform due to lack of familiarity, and the atypical nature of conversing and engaging through virtual means, particularly where issues with poor broadband connection had occurred. An in-person or hybrid approach would have possibly enhanced opportunities for urban design and planning—for example, through the use of 3-D models, constructing spaces and places and mapping and other co-creation opportunities that work more effectively through face-to-face interaction. In terms of the transnational nature of the study, although the purpose of this knowledge co-creation study was not focused on enabling cross-cultural comparability but rather to bring together important and varied cross-cultural knowledges toward mutual benefit and impact; an analysis of the various ways in which the recommendations and solutions could be applied in different socio-cultural contexts would have been beneficial.

Regarding the INTERGEN survey, though designed with the intent to acquire rich qualitative data, the analysis would have benefited from including validated scales, that could have provided a better snapshot of the distribution and intergenerational determinants that shape health and wellbeing outcomes. To this effect, it is important to note that the INTERGEN survey did not adhere to a traditional quantitative paradigm (i.e., no sampling frame, no probabilistic sample and no statistical analysis) which may have rendered the survey results to be of low validity. Last, as the participants consisted of a convenience sample of individuals who were interested in the topic area and were willing to participate, there is the potential that the sample largely included the perspectives of those who shared similar interests and opinions.

### 4.3. Concluding remarks

To co-create opportunities for developing mutually beneficial spaces is a substantial undertaking that requires cross-sectoral and cross-disciplinary working and prioritizing community and lay perspectives in the development and decision-making process. There is a crucial need for more coherent ways for ensuring that people of all ages are integrated into the matrix of opportunities afforded by local and digital communities and benefiting from both national and international aging initiatives for living well in later life. The AFLE project has resulted in the conceptualization of an intergenerational and age-friendly eco-system to support and provide opportunities for people as they age to maximize the socioeconomic benefits of their local and virtual communities and help them become fully integrated, valued and contributory members of society. The next phase of the project will include efforts for developing an upscaled, longitudinal, multi-site, multi-national transdisciplinary research initiative toward collectively building an intergenerational and age-friendly living ecosystem.

## Data availability statement

The raw data supporting the conclusions of this article will be made available by the authors, without undue reservation.

## Author contributions

Project conception and design and data collection: MLF, JS, and AH-P. Project instrument design: MLF, JS, AH-P, PS, and RP. Analysis and interpretation of results: RR, JS, MLF, AH-P, PS, RP, RW, and RC. Draft manuscript and preparation: MLF, RR, and JS. Critical revision of manuscript for important intellectual content: MLF, JS, AH-P, PS, RP, RW, and RC. All authors reviewed the results and approved the final version of the manuscript.

## References

[1] United Nations. World Population Ageing 2017—Highlights. New York: United Nations, Department of Economic and Social Affairs, Population Division (2017).

[2] World Health Organization. Towards an Age-friendly World. (2019). Available online at: https://www.who.int/ageing/age-friendly-world/en/ (accessed January 16, 2020).

[3] World Health Organization. World Report on Ageing and Health. Geneva: World Health Organization (2015).

[4] Fang ML Canham SL Battersby L Sixsmith J Wada M Sixsmith A. Exploring privilege in the digital divide: implications for theory, policy, and practice. Gerontologist. (2018) 9:e1–e15. 10.1093/geront/gny037 29750241

[5] World Health Organization. Global Age-Friendly Cities Project. (2019). Available online at: https://www.who.int/ageing/projects/age_friendly_cities/en/ (accessed January 16, 2020).

[6] D'Cruz M Banerjee D. An invisible human rights crisis: the marginalization of older adults during the COVID-19 pandemic—an advocacy review. Psychiatry Res. (2020) 292:113369. 10.1016/j.psychres.2020.113369 32795754 PMC7397988

[7] Fang ML Walker M Wong KLY Sixsmith J Remend L Sixsmith A. Future of Digital Health and Community Care: Exploring Intended Positive Impacts and Unintended Negative Consequences of COVID-19. Healthcare Management Forum. (2022). Los Angeles, CA: SAGE Publications. 10.1177/08404704221107362 PMC9253718 35775162

[8] Kaplan M Sanchez M Hoffman J. Intergenerational Pathways to a Sustainable Society. Cham: Springer International Publishing (2017). 10.1007/978-3-319-47019-1

[9] World Health Organization. Global Age-Friendly Cities: A Guide. Geneva: WHO Press (2007).

[10] Van Hoof J Kazak JK Perek-Bialas JM Peek STM. The challenges of urban ageing: making cities age-friendly in Europe. Int J Environ Res Public Health. (2018) 15:2473. 10.3390/ijerph15112473 30714577 PMC6266083

[11] Ng KYY Leung GYC Tey AJ Chaung JQ Lee SM Soundararajan A. Bridging the intergenerational gap: the outcomes of a student-initiated, longitudinal, inter-professional, inter-generational home visit program. BMC Med Educ. (2020) 20:148. 10.1186/s12909-020-02064-x 32393249 PMC7216381

[12] Kirkpatrick MK Brown ST. Leadership development in geriatric care through the intergeneration make a difference project. Nurs Educ Perspect. (2006) 27:89–92. 16733972

[13] Frieden TR. The future of public health. New England J Med. (2015) 373:1748–54. 10.1056/NEJMsa1511248 26510022

[14] Haldane V De Foo C Abdalla SM Jung A-S Tan M Wu S. Health systems resilience in managing the COVID-19 pandemic: lessons from 28 countries. Nat Med. (2021) 27:964–80. 10.1038/s41591-021-01381-y 34002090

[15] Woolrych R Sixsmith J Duvvuru J Portella A Fang ML Menezes D. Cross-national perspectives on aging and place: implications for age-friendly cities and communities. Gerontologist. (2022) 62:119–29. 10.1093/geront/gnab170 34791252

[16] United Nations. Transforming Our World: The 2030 Agenda for Sustainable Development. (2019). Available online at: https://sustainabledevelopment.un.org/post2015/transformingourworld (accessed January 16, 2020).

[17] Grigorovich A Fang ML Sixsmith J Kontos P. Defining and evaluating the effectiveness of transdisciplinary research in aging and technology. Disabil Rehabil Assist Technol. (2019) 14:533–42. 10.1080/17483107.2018.1496361 30318930

[18] Boger J Jackson P Muvenna M Sixsmith J Sixsmith A Mihailidis A. Principles for fostering the transdisciplinary development of assistive technologies. Disabil Rehabil Assist Technol. (2017) 12:480–90. 10.3109/17483107.2016.1151953 27052793

[19] Rittel HW Webber MM. Dilemmas in a general theory of planning. Policy Sci. (1973) 4:155–69. 10.1007/BF01405730

[20] Evans J. Ideational border crossings: rethinking the politics of knowledge within and across disciplines. Discourse Stud Cult Politics Edu. (2014) 35:45–60. 10.1080/01596306.2012.739466

[21] Jagosh J Bush PL Salsberg J Macaulay AC Greenhalgh T Wong G. A realist evaluation of community-based participatory research: partnership synergy, trust building and related ripple effects. BMC Public Health. (2015) 15:1. 10.1186/s12889-015-1949-1 26223523 PMC4520009

[22] Ward V Smith S Foy R House A Hamer S. Planning for knowledge translation: a researcher's guide. Evidence Policy J Res Debate Practice. (2010) 6:527–41. 10.1332/174426410X535882

[23] Maasen S Lieven O. Transdisciplinarity: a new mode of governing science? Sci Public Policy. (2006) 33:399–410. 10.3152/147154306781778803

[24] Kang HK. “We're who we've been waiting for”: intergenerational community organizing for a healthy community. J Commun Practice. (2015) 23:126–40. 10.1080/10705422.2014.983214

[25] Centre for Ageing Better,. Age-Friendly Communities. (2021). Available online at: https://ageing-better.org.uk/age-friendly-communities (accessed September 8, 2021).

[26] Government of UK Department for Business EIS. Policy Paper: The Grand Challenge Missions. (2021). Available online at: https://www.gov.uk/government/publications/industrial-strategy-the-grand-challenges/missions#ageing-society (accessed September 8, 2021).

[27] Woolrych R Sixsmith J Fisher J Makita M Lawthom R Murray M. Constructing and negotiating social participation in old age: experiences of older adults living in urban environments in the United Kingdom. Ageing Soc. (2019) 41:1398–420. 10.1017/S0144686X19001569

[28] Fang ML Sixsmith J Lawthom R Mountian I Shahrin A. Experiencing 'pathologized presence and normalized absence'; understanding health related experiences and access to health care among Iraqi and Somali asylum seekers, refugees and persons without legal status. BMC Public Health. (2015) 15:923. 10.1186/s12889-015-2279-z 26386559 PMC4575487

[29] Kaplan M Thang LL Sanchez M Hoffman J. Intergenerational Contact Zones: Place-based Strategies for Promoting Social Inclusion and Belonging. New York: Routledge (2020). 10.4324/9780429199097

[30] Guo Y Liu Y Lu S Chan OF Chui CHK Lum TYS. Objective and perceived built environment, sense of community, and mental wellbeing in older adults in Hong Kong: a multilevel structural equation study. Landsc Urban Plan. (2021) 209:104058. 10.1016/j.landurbplan.2021.104058

[31] Tang JY Chui CH Lou VW Chiu RL Kwok R Tse M. The contribution of sense of community to the association between age-friendly built environment and health in a high-density city: a cross-sectional study of middle-aged and older adults in Hong Kong. J Appl Gerontol. (2021) 40:1687–96. 10.1177/0733464821991298 33554751

[32] Cushing DF van Vliet W. Intergenerational communities as healthy places for meaningful engagement and interaction. Fam Intergenerational Peer Gr Relat. (2016) 5:239–65. 10.1007/978-981-287-026-1_10

[33] Bronfenbrenner U. Ecological Systems Theory. London: Jessica Kingsley Publishers (1992).

[34] Vindigni D Jones S. Integrated Pathways to Healthy Ageing (PHA): A Conceptual Ecosystem. Mill Park, Asutralia: RMIT University Bundoora Campus (2020). 10.21203/rs.3.rs-37011/v1

[35] Noble H Heale R. Triangulation in research, with examples. Evid Based Nurs. (2019) 22:67–8. 10.1136/ebnurs-2019-103145 31201209

[36] Bager T. The camp model for entrepreneurship teaching. Int Entrepreneurship Manage J. (2011) 7:279–96. 10.1007/s11365-010-0149-9

[37] Braun V Clarke V. Reflecting on reflexive thematic analysis. Qualitative Res Sport Exercise Health. (2019) 11:advanced online e-pub. 10.1080/2159676X.2019.1628806

[38] Byrne D A. worked example of Braun and Clarke's approach to reflexive thematic analysis. Qual Quant. (2022) 56:1391–412. 10.1007/s11135-021-01182-y

[39] Boyatzis RE. Transforming Qualitative Information: Thematic Analysis and Code Development. London, UK: Sage Publications Ltd. (1998).

[40] Canham SL Fang ML Battersby L Woolrych R Sixsmith J Ren TH. Contextual factors for aging well: creating socially engaging spaces through the use of deliberative dialogues. Gerontologist. (2018) 58:140–8. 10.1093/geront/gnx121 28977373

[41] Fang ML Sixsmith J Hamilton-Pryde A Rogowsky R Scrutton P. Intergenerational and Age-friendly Living Ecosystems (AFLE). Dundee, Scotland, United Kingdom: University of Dundee (2022). 10.20933/100001223 PMC9846501 36685002

[42] Hooks B. Feminist Theory: From Margin to Center. 2nd ed. Brooklyn: South End Press (2000).

[43] Heu LC van Zomeren M Hansen N. Lonely alone or lonely together? A cultural-psychological examination of individualism–collectivism and loneliness in five European countries. Personal Soc Psychol Bull. (2019) 45:780–93. 10.1177/0146167218796793 30264659 PMC6449799

[44] Polk M. Transdisciplinary co-production: designing and testing a transdisciplinary research framework for societal problem solving. Futures. (2015) 65:110–22. 10.1016/j.futures.2014.11.001

[45] Buffel T De Backer F Peeters J Phillipson C Reina VR Kindekens A. Promoting sustainable communities through intergenerational practice. Proc Soc Behav Sci. (2014) 116:1785–91. 10.1016/j.sbspro.2014.01.47228865659

[46] Steinfeld E Maisel J. Universal Design: Creating Inclusive Environments. Hoboken: John Wiley & Sons (2012).

[47] Pratesi A Sixsmith J Woolrych R. Genuine partnership and equitable research: working “with” older people for the development of a smart activity monitoring system. Innovat J. (2013) 18:2–17.

[48] Sixsmith J Fang ML Woolrych R Canham SLB Sixsmith A. Ageing well in the right place: partnership working with older people. Working Older People. (2017) 21:40–8. 10.1108/WWOP-01-2017-0001

[49] Jones L Wells K. Strategies for academic and clinician engagement in community-participatory partnered research. JAMA. (2007) 297:407–10. 10.1001/jama.297.4.407 17244838

[50] Graham ID Kothari A McCutcheon C. Moving knowledge into action for more effective practice, programmes and policy: protocol for a research programme on integrated knowledge translation. Implement Sci. (2018) 13:1–15. 10.1186/s13012-017-0700-y 29394932 PMC5797415

