# Supplementary material for: Co-creating inclusive spaces and places: Towards an intergenerational and age-friendly living ecosystem
Source: Front Public Health. 2023 Jan 4;10:996520. doi: 10.3389/fpubh.2022.996520 (PMC9846501; doi:10.3389/fpubh.2022.996520)
Supplement: Supplementary file 1 [file Table_1.DOCX]

Supplementary Material

# Supplementary Figures and Tables

For more information on Supplementary Material and for details on the different file types accepted, please see [here](http://home.frontiersin.org/about/author-guidelines#SupplementaryMaterial). Figures, tables, and images will be published under a Creative Commons CC-BY licence and permission must be obtained for use of copyrighted material from other sources (including re-published/adapted/modified/partial figures and images from the internet). It is the responsibility of the authors to acquire the licenses, to follow any citation instructions requested by third-party rights holders, and cover any supplementary charges.

## Supplementary Figures


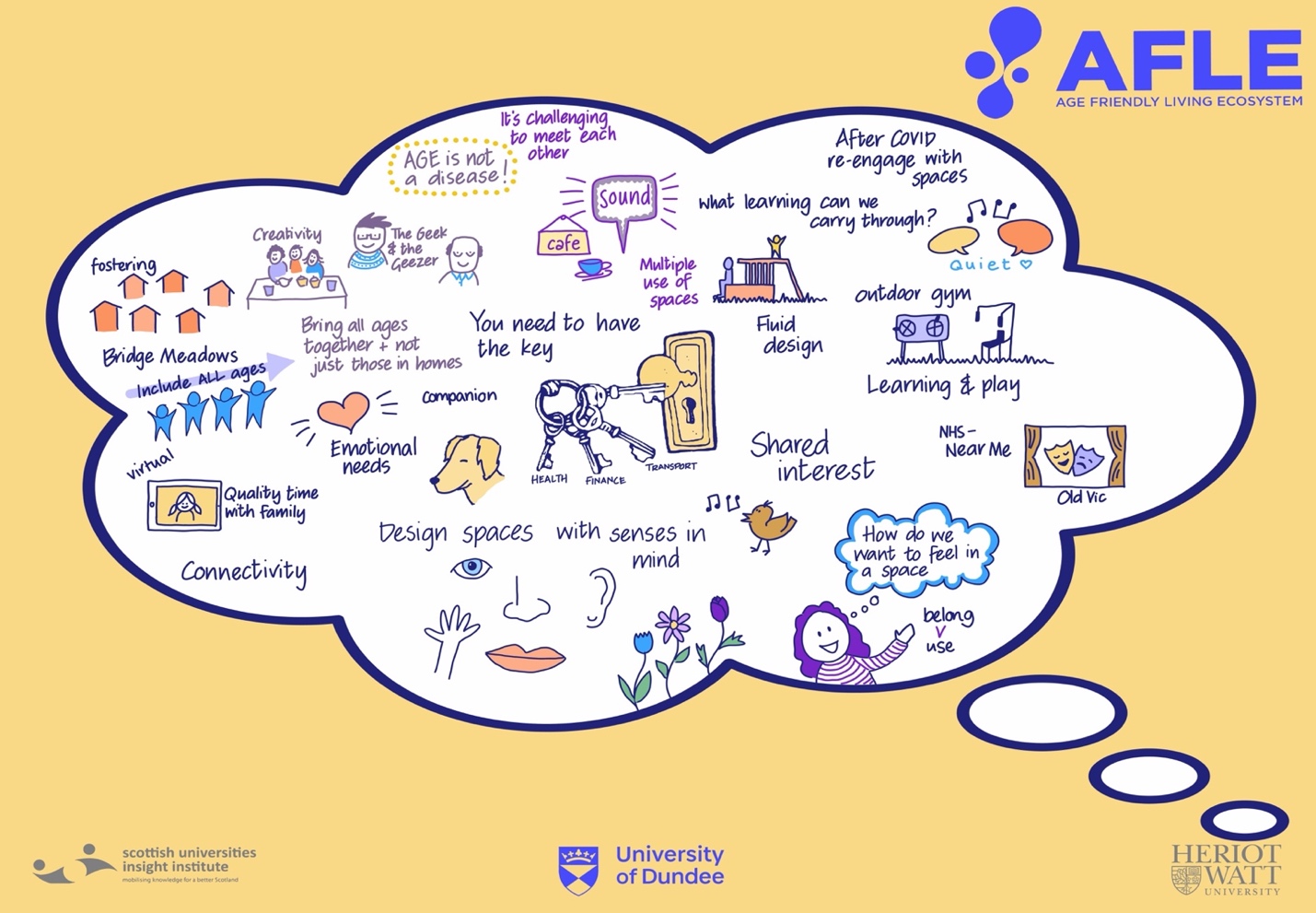


**Supplementary Figure 1.** Conceptual map of ideas towards an intergenerational and age-friendly living ecosystem. Image was produced by Clare Mills, a graphic facilitator who was commissioned to join the virtual co-creation sessions to capture notes and ideas in illustrative form.

**
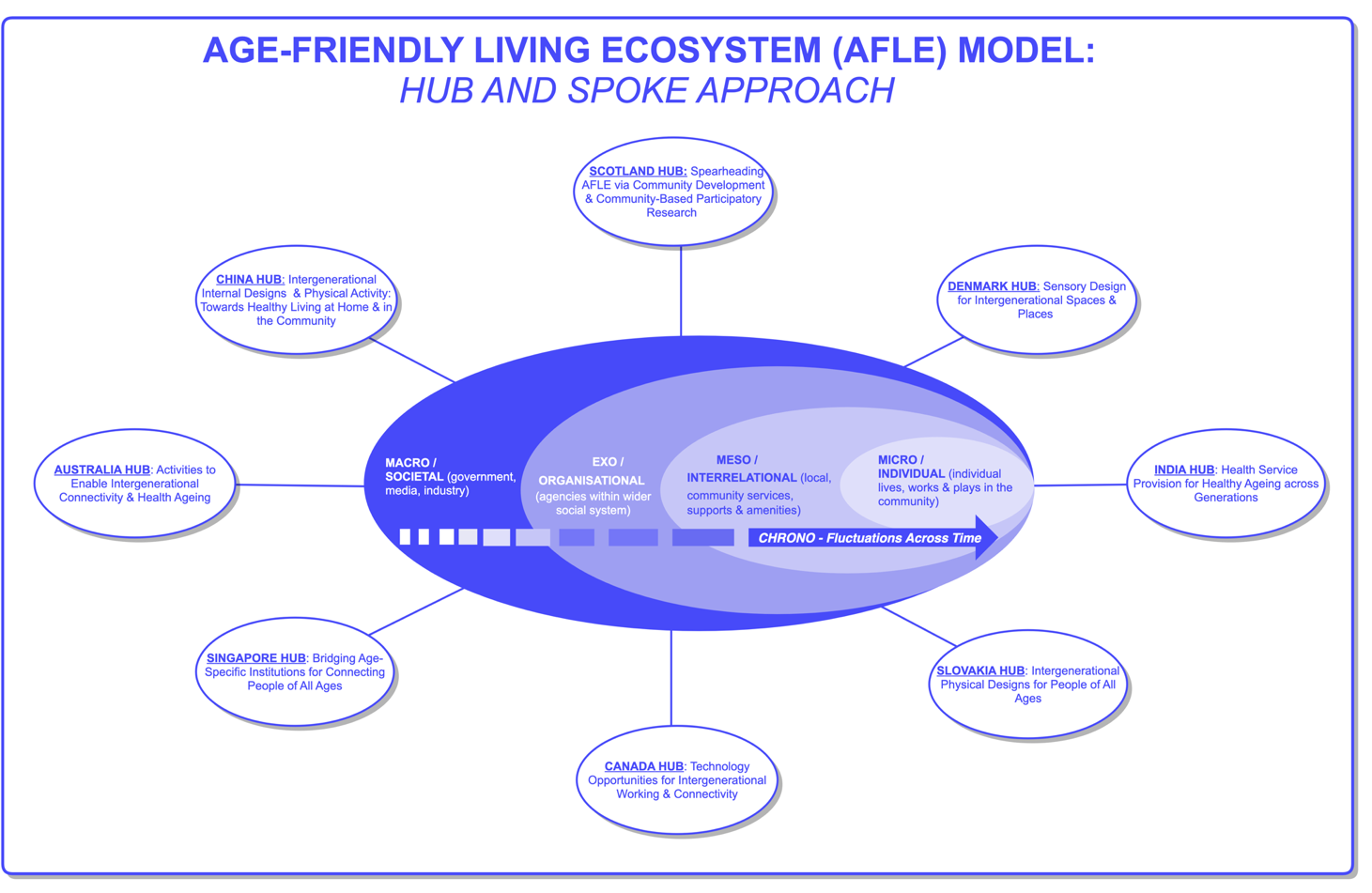
**

**Supplementary Figure 2.** A conceptual model co-produced by the project team using the Gliffy diagram tool which provides the vision for an age-friendly living ecosystem with collaborating countries.

**
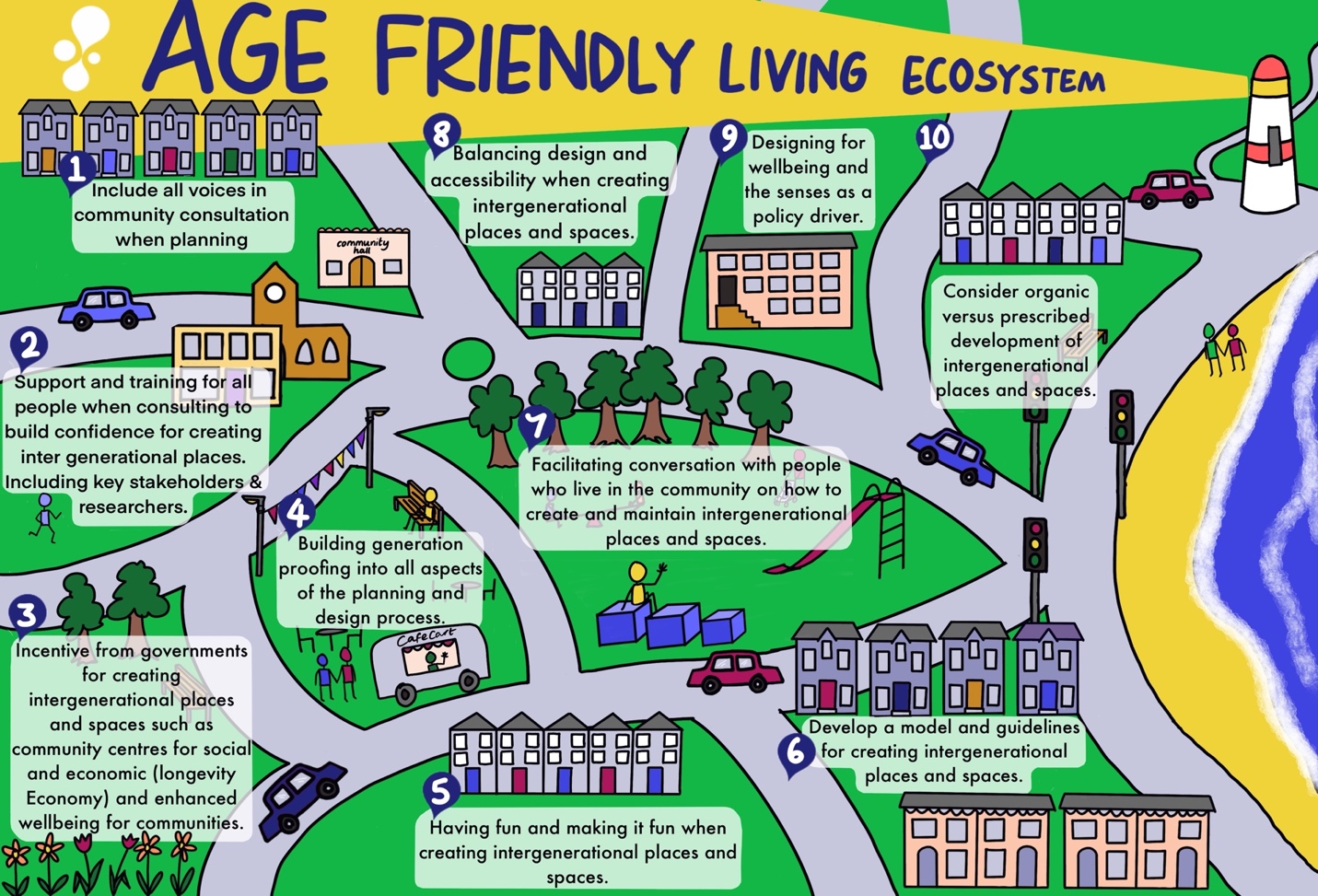
**

**Supplementary Figure 3.** A policy road map of ideas for the development of an intergenerational and age-friendly living ecosystem. Image was produced by Clare Mills, a graphic facilitator who was commissioned to join the virtual co-creation sessions to capture notes and ideas in illustrative form.

## Supplementary Tables

**Supplementary Table 1.** INTERGEN survey questions.

| **INTERGEN Survey Instructions** |
| --- |
| We, the project team, are trying to find out how best to design age-friendly intergenerational places. These are places where people of all ages feel comfortable and enjoy time together, interact and actively participate in community activities. They are places that treat everyone with respect and make it easy for older people to stay connected to people that are important to them. This will help the project team to put together guidelines to support the development of age-friendly intergenerational places.  Answer as many questions as you can and as honestly as you can.  Tell us what you think, there are no right or wrong answers.  The more detailed your answers, the more helpful they are.  **In the following questions:**  **An age-friendly place** enables people of all ages to actively participate in community activities and treats everyone with respect, regardless of their age. It is a place that makes it easy for older people to stay connected to people that are important to them.  **Intergenerational places** mean places where people of all ages feel comfortable to be together, to enjoy time together and interact |
| **Demographic Questions: Can you please tell us a little bit about you** |
| 1. How old are you?  - 12 and under - 13-17 - 18-24 - 25-34 - 35-44 - 45-54 - 55-64 - 65-74 - 75-84 - 85 and over  1. How would you describe your gender?  - [ ] Male (including transgender men) - [ ] Female (including transgender women) - [ ] Prefer to self-describe as (non-binary, gender-fluid, agender, please specify) - [ ] Prefer not to say - [ ] Other  1. Please indicate the country in which you reside (drop down list of countries) |
| **INTERGEN Items** |
| 1. In normal circumstances, how often do you interact with people who are: younger than you (outside the immediate family)? **Often, Sometimes, Seldom, Never** 2. In normal circumstances, how often do you interact with people who are: older than you (outside the immediate family)? **Often, Sometimes, Seldom, Never** 3. What makes a place ‘age-friendly’? List the main features of an age-friendly place. 4. What are the features of an ‘intergenerational’ place? List the main features of an intergenerational place. 5. What makes a place attractive for younger people? INFORMATION: Children and young people up to age 25. 6. What makes a place attractive for older people? INFORMATION: People wo are over 60 years. 7. What makes a place feel safe for younger people? INFORMATION: Children and young people up to age 25. 8. What makes a place feel safe for older people? INFORMATION: People wo are over 60 years. 9. Have you been to a space that you consider to be ‘intergenerational’ before? (YES/NO)    1. If yes, please describe it. 10. What sensory qualities (e.g. how it looks, what it feel like, etc.) do you think are important within intergenerational places. 11. Who should be involved in creating intergenerational places? 12. Please use the space below to share your final thoughts and ideas that sum up what an intergenerational place might be like. 13. What do you think are the barriers to young people's engagement in intergenerational places? 14. What do you think are the barriers to older people's engagement in intergenerational places? 15. If you would like to be a part of the AFLE Network, please provide your name and email address. We will add you to our AFLE Network Repository. |
| **Survey Completed** |
| Thank you very much for participating. We appreciate your input! |
